# A think-aloud study exploring the application of composite time trade-off and discrete choice experiment methods for valuing the Chinese Short Warwick-Edinburgh Mental Well-being Scale (C-SWEMWBS)

**DOI:** 10.1186/s12955-025-02433-4

**Published:** 2025-10-23

**Authors:** Hei Hang Edmund Yiu, Ling Hin Chow, Cheri Cheuk Lam Au, Eunice Kehui Deng, Zimeng Zhao, Yue Wei, Kyung Jin Lee, Caige Huang, Yu Yang, Wei Kang, Stavros Petrou, Jason Madan, Esther W. Chan

**Affiliations:** 1 Centre for Safe Medication Practice and Research, Department of Pharmacology and Pharmacy, LKS Faculty of Medicine, The University of Hong Kong, Hong Kong SAR, China; 2 Laboratory of Data Discovery for Health (D24H), Hong Kong Science and Technology Park, Hong Kong SAR, China; 3 Nuffield Department of Primary Care Health Sciences, University of Oxford, Oxford, UK; 4 Centre for Health Economics at Warwick, Warwick Medical School, University of Warwick, Coventry, UK; 5 Department of Pharmacy, The University of Hong Kong-Shenzhen Hospital, Shenzhen, China; 6 The University of Hong Kong Shenzhen Institute of Research and Innovation, Shenzhen, China

**Keywords:** C-SWEMWBS, Think-aloud, Cognitive interview, Composite time trade-off, Discrete choice experiment

## Abstract

**Background:**

To investigate the cognitive experiences of completing composite time trade-off (C-TTO) and discrete choice experiment (DCE) tasks for the valuation of the Chinese Short Warwick-Edinburgh Mental Well‐being Scale (C-SWEMWBS) in Hong Kong in order to inform an appropriate preference elicitation protocol for this population.

**Methods:**

Eighteen think-aloud interviews employing concurrent and retrospective think-aloud techniques were conducted with Cantonese-speaking adult members of the general population. Each participant completed five C-TTO and five DCE tasks with tailor-made C-SWEMWBS states. Interview transcripts were transcribed verbatim, translated into English, and analysed thematically to identify patterns in participants’ thoughts and understanding during task completion.

**Results:**

Participants generally found the C-TTO and DCE tasks manageable. Four themes were identified that reflected participants’ experiences: (1) Interview design and structure, bringing attention to potential refinements in presentation and instructions; (2) Representation of items and levels, illustrating participants’ comprehension of the different C-SWEMWBS items and levels; (3) Influences on decision-making, identifying personal and external factors that shaped participant’s decisions; and (4) Appropriateness of measures, reflecting participant’s experiences in imagining and deriving utility values for mental well-being states.

**Conclusion:**

Despite highlighting areas that could be refined to minimise unnecessary cognitive burden, findings indicate that the design of the C-TTO and DCE tasks is both feasible and appropriate for the preference elicitation of C-SWEMWBS states in Hong Kong. This provides the basis for conducting a large-scale study to derive a preference-based value set for the C-SWEMWBS in Hong Kong for economic evaluations of interventions aimed at improving public mental well-being.

**Supplementary Information:**

The online version contains supplementary material available at 10.1186/s12955-025-02433-4.

## Introduction

The Mental Well-being Adjusted Life Year (MWALY) is a metric used for cost-utility analyses of interventions aimed at improving mental well-being (MWB) [1, 2]. The MWALY, which combines MWB quality-adjustment weights with the change in life years associated with an intervention, serves as the effectiveness component in the incremental cost-effectiveness ratio (ICER). The ICER estimates the additional cost per MWALY gained, enabling comparison of value for money across interventions and informing decisions under budget constraints. Estimating MWALYs requires a preference-based measure of MWB, which can be developed by generating a value set for an existing MWB instrument. The process of generating a value set can be based on surveys of individuals with the values elicited through stated preference methods [2, 3].

The Short Warwick-Edinburgh Mental Well-being Scale (SWEMWBS) is a widely used MWB measure developed in the UK, validated across various jurisdictions with strong internal consistency and reliability [4–6]. The Chinese-translated version of the SWEMWBS (C-SWEMWBS) has shown good construct validity and test-retest reliability in Hong Kong [7, 8]. The questionnaire comprises seven positively worded items assessing MWB, each with five levels spanning from “none of the time” to “all of the time”. Developed from hedonic and eudaimonic perspectives of well-being, where hedonic refers to subjective well-being focused on feelings, moods, and the balance of pleasure over pain, and eudaimonic refers to well-being focused on achieving self-actualisation, the items capture aspects targeted by many public mental health interventions, making it suitable for developing a preference-based MWB valuation tool [2, 8–10].

Stated preference elicitation methods, particularly composite time trade-off (C-TTO) and discrete choice experiment (DCE), are widely used to generate value sets for generic preference-based measures, including the EuroQol five-dimensional five-level (EQ-5D-5L) questionnaire and the SWEMWBS, to estimate Quality-Adjusted Life Years (QALYs) or MWALYs [2, 11, 12]. Drawing on the concept of opportunity cost, the C-TTO task involved two living alternatives with varying durations of MWB states. A conventional TTO method was used for SWEMWBS states considered better-than-dead, where participants determined the indifference point between Life A (𝑥 years of full MWB) and Life B (10 years in a designated MWB state) through trading off years in Life A [13]. A lead-time TTO method was used for SWEMWBS states considered worse-than-dead, where participants determined the indifference point between Life A (𝑥 years of full MWB) and Life B (10 years of full MWB followed by 10 years in a designated MWB state) through trading off years in Life A. Based on random utility theory, the DCE task involved pairwise forced-choice comparisons of SWEMWBS items and levels [14]. Yiu et al. demonstrated the feasibility of C-TTO and DCE methods for valuing SWEMWBS in the UK [10]. A subsequent study derived a SWEMWBS value set using C-TTO and DCE methods within the UK population, establishing SWEMWBS as a preference-based MWB measure in the UK [2]. Yet, the C-SWEMWBS lacks an established preference-based value set for Hong Kong. Notably, the UK SWEMWBS valuation study showed differences relevant to mental versus physical health state valuation. Specifically, some participants exhibited non-monotonic preferences, meaning they did not view full MWB (the highest level on all SWEMWBS items) as the most desirable outcome. Such differences can affect how trade-offs are interpreted and how values are modelled [2].

Concurrently, evidence highlights the necessity of jurisdiction-specific SWEMWBS value sets for cost-utility analysis. Value sets differ between societies and cultures [15]. For instance, the EQ-5D has established value sets for various jurisdictions, including England and Hong Kong [15, 16]. The EQ-5D-5L value set for England has a minimum of − 0.285 with about 5% of health states valued as worse-than-dead, whereas Hong Kong’s value set has a minimum of approximately − 0.864 with about 36% of states worse-than-dead [12, 16]. These contrasts likely reflect societal preferences regarding tolerance for extreme functional impairment and suffering, and they imply larger health gains from moving patients out of very severe states under the Hong Kong value set. This underscores the need for jurisdiction-specific value sets. Additionally, understandings of well-being differ culturally [12, 17]. Given known cross-cultural differences in well-being constructs and beliefs, and established jurisdiction-specific variation in value sets, it cannot be assumed that hedonic and eudemonic perspectives fully encapsulate how the Hong Kong population defines ideal MWB, nor that SWEMWBS utility values derived from the UK population generalise to Hong Kong. As the use of C-TTO and DCE for valuing the C-SWEMWBS has not been explored in Hong Kong, it remains uncertain whether these two methods are feasible for eliciting valuations of MWB states in this context.

The think-aloud technique, where participants verbalise their thoughts and feelings while processing information [18], offers a viable tool to explore the appropriateness of preference-based valuation protocols through gathering cognitive experiences of completing health preference tasks [19–21]. This study aims to investigate the cognitive experiences of completing C-TTO and DCE tasks for the valuation of C-SWEMWBS in Hong Kong using the think-aloud technique. The findings will identify task features, item and level interpretations, and decision strategies specific to this context. These insights are necessary to confirm feasibility for MWB valuation in Hong Kong, to refine the valuation protocol to minimise bias and cognitive burden, and to support the development and valid interpretation of a Hong Kong specific C-SWEMWBS value set for MWALY estimation.

## Methods

Think-aloud interviews were conducted either in person or via Zoom with Cantonese-speaking adults aged 18 years or above from the general population in Hong Kong. Participants were recruited using convenience and purposive sampling through personal networks and social media to ensure demographic diversity in terms of age, gender, occupation, and education. The sample size was initially set at 12 and finalised based on data saturation, determined when three consecutive interviews yielded no new informative themes [22]. The interviewer completed structured, point‑form field notes in a pre‑formatted Word table for each interview. The table captured patterns and problems at each stage and grouped entries under task comprehension, decision strategies, responses to options, and other difficulties. A dedicated section flagged any new informative messages relative to prior interviews. Each participant received a HKD100 supermarket coupon upon completing the interview. This study was approved by the Institutional Review Board of the University of Hong Kong/Hospital Authority Hong Kong West Cluster (UW 23–204).

**Fig. 1:**
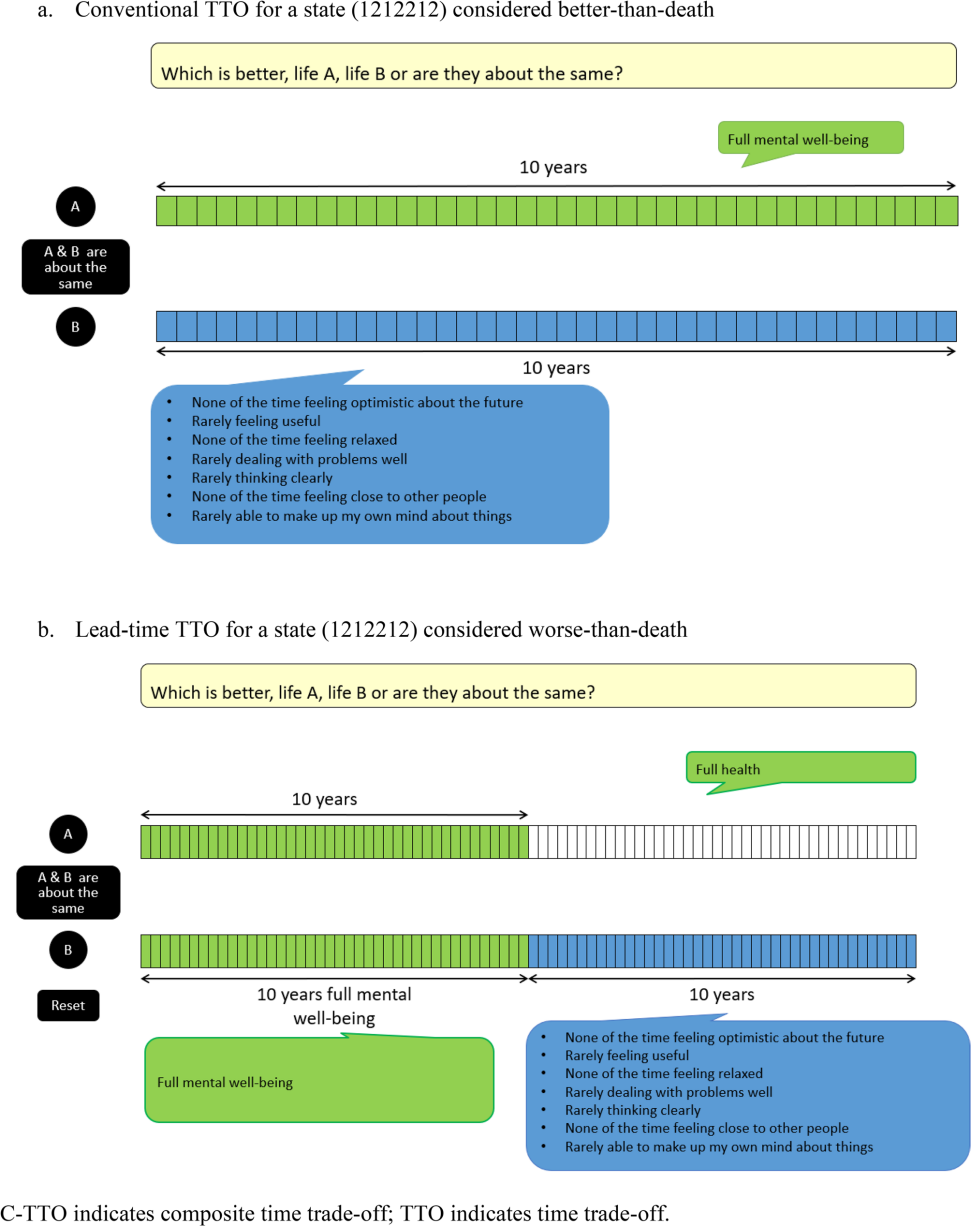
Example of a C-TTO task

**Fig. 2:**
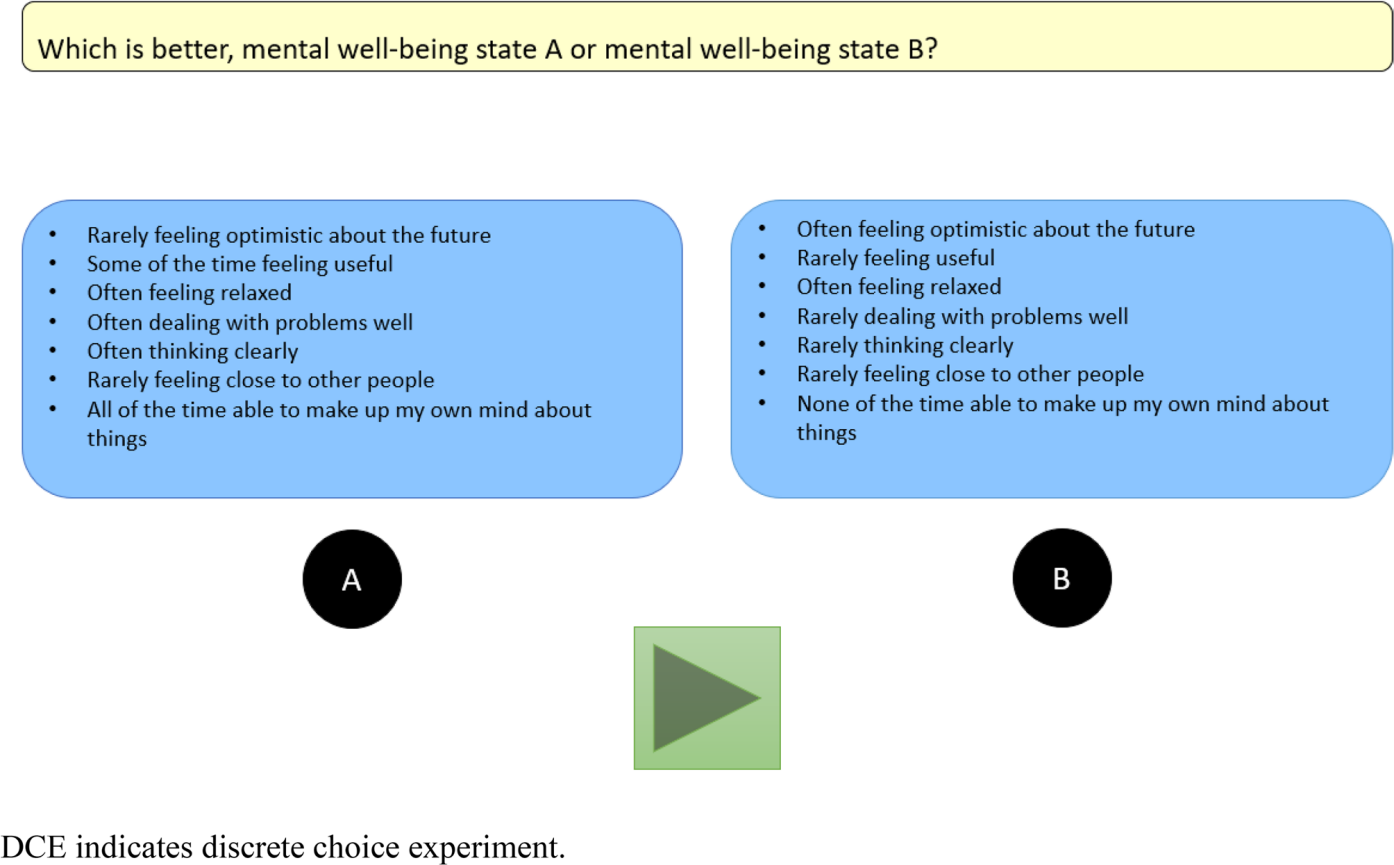
Example of a DCE task (2344425 versus 4242221)

Participants verbalised their feelings and thoughts about completing the C-TTO and DCE tasks, supplemented by probing questions. These tasks were administered via one-to-one computer-assisted personal interviewing using the EuroQol Portable Valuation Technology (EQ-PVT) platform embedded in PowerPoint, which randomised the order of the tasks displayed to each participant [23]. Examples of C-TTO and DCE tasks are illustrated in Figs. 1 and 2.

### Design of the C-TTO and DCE tasks

Each participant completed five C-TTO tasks and five DCE tasks, covering states with varying levels of MWB (Table 1). For C-TTO, a state closer to full MWB (5454555) was included to examine the proportion of participants unwilling to trade off years of full MWB to avoid this state or preferring this state over full MWB, as observed in previous SWEMWBS valuation studies conducted in the UK [2]. The lowest MWB state (1111111) was included to assess the possible minimum C-TTO value assigned by the participants. Intermediate MWB states were designed to explore the importance of relationships with others in evaluating the overall state, as prior SWEMWBS valuation studies found that the attribute related to feeling close to other people was the most highly valued by the general population [2].

**Table 1:** Design of the C-TTO and DCE States

*C-TTO*			
**State number**	**States**	**Relative mental well-being level**	**Purpose of the state**
TTO1	5454555	High	To explore whether non-trading effects or non-monotonic preferences are found in states closer to full mental well-being.
TTO2	4343353	Intermediate	To explore how participants interpret a state with an intermediate level of mental well-being, focusing on a high level of feeling close to others.
TTO3	3434424	Intermediate	To explore how participants interpret a state with an intermediate level of mental well-being, focusing on a low level of feeling close to others.
TTO4	1212212	Low	To explore how participants interpret a state with an overall low level of mental well-being.
TTO5	1111111	Low	To observe how many years of full mental well-being participants are willing to give up to avoid being in this lowest mental well-being state.
***DCE***			
**Pair number**	**Pairs of comparison**	**Purpose of the state**	
DCE1	5555555 versus 3333333	A monotonicity test to explore whether participants’ choices are rational.	
DCE2	2423525 versus 3342454	To explore whether participants value feeling closer to others more.	
DCE3	1112232 versus 2221313	To explore whether participants tend to avoid the lowest levels of attributes and engage in tallying.	
DCE4	2252254 versus 3333333	To explore whether participants prefer a balanced state.	
DCE5	2344425 versus 4242221	To explore whether overlapping attributes (often feeling relaxed and rarely feeling close to other people) reduce cognitive burden when completing the task.	

C-TTO indicates composite time trade-off; TTO indicates time trade-off; DCE indicates Discrete Choice Experiment

For the DCE, choice pairs were designed to investigate decision-making rationality, the role of relationships, and the impact of fewer attributes with varying levels on reducing trade-off burden. The design also explored common decision-making strategies identified in previous SWEMWBS valuation studies, such as avoiding extremely low levels, tallying, and preferring a balanced state across attributes, among others [10].

### Interview procedure

All interviews were recorded and guided by an interviewer in a computer-assisted personal interview setting. The procedure included:

1. The interviewer introduced the study background.
2. The participant signed an informed consent form.
3. The participant completed a demographics form and the C-SWEMWBS describing their own MWB.
4. The participant completed a think-aloud “window counting” warm-up exercise [24].
5. For in-person interviews, the interviewer used a laptop with EQ-PVT installed and displayed the task screens to the participant. For online interviews conducted via Zoom, the interviewer shared the screen and granted the participant remote-control access so that the participant could interact with the tasks directly.
6. C-TTO tasks: The participant was guided through an initial practice scenario involving a MWB state related to a lack of confidence and self-esteem (Supplementary Information 1). Two dynamic practice questions followed, covering scenarios better and worse than the initial state, to familiarise the participant with different evaluative spaces. The participant stated preferred options aloud while the interviewer clicked on the participant’s behalf to document the trade-off process. The participant then completed three additional practice C-SWEMWBS states representing varying levels of MWB: 4554545 (high), 4212354 (intermediate), and 2111131 (low). For in-person interviews, the participant used a mouse to make selections; for online interviews via Zoom, the participant clicked directly using remote control. The participant iteratively adjusted the time in full MWB to indicate preferences, could reverse choices when needed, and continued until reaching indifference between Life A and Life B for each task. The bisection approach was used for the first three steps, followed by upward or downward titration with one-year or six-month adjustments [25]. A reset button was available to restart a task. After the practice tasks, the participant completed the five C-TTO tasks. To minimise recall bias and cognitive fatigue, concurrent think-aloud techniques were used during the first three tasks, while retrospective think-aloud techniques were adopted for the last two. After task completion, a feedback slide showed the rank ordering of the five states inferred from the participant’s responses. The participant indicated and explained any disagreement with the ranking of states. Finally, debriefing questions explored the participant’s decision-making rationale (Supplementary Information 2).
7. DCE tasks: The participant selected the preferred MWB alternative directly, using a mouse in person or via remote control on Zoom. Concurrent think-aloud techniques were applied to the first three tasks and retrospective techniques to the remaining two. Debriefing questions were asked (Supplementary Information 3).

### Data analysis

All interviews were transcribed verbatim in traditional Chinese and translated into English. Two bilingual researchers independently reviewed the translations for accuracy, resolving discrepancies to ensure linguistic and cultural fidelity. A thematic analysis was conducted to interpret latent meanings and generate higher-order themes [26–28]. Initially, open coding was performed by two independent reviewers on the first five transcripts to summarise key information, supplemented by written interview field notes. A third reviewer resolved any discrepancies in interpretation. A coding tree was then developed to identify potential codes and themes and establish relationships among them. A codebook documented the underlying meanings of the codes and themes. Using this framework, the first five transcripts were coded in NVivo 14 by the first and second reviewers. The third reviewer checked the coding results to refine the framework by combining, adding, or removing codes. The updated framework was applied to the remaining transcripts, which were coded by the first reviewer and checked by the second and third reviewers for robustness. A descriptive account categorised codes and themes, supported by representative quotes from the interviews.

## Results

Eighteen interviews were conducted between 14 October 2023 and 8 February 2024, with three face-to-face, and the remainder via Zoom. No new informative messages emerged in interviews 16–18, indicating saturation. On average, each interview lasted 104.9 min (standard deviation [SD] = 31.2). Table 2 summarises the participants’ demographic characteristics, demonstrating diversity in educational backgrounds and income levels, with a mean age of 44.8 years (SD = 16.5) and a mean C-SWEMWBS score of 25.9 (SD = 3.1). Four themes were identified to capture participants’ cognitive experiences of completing C-TTO and DCE tasks for valuing the C-SWEMWBS (Table 3).

**Table 2:** Demographic characteristics of the 18 participants

Characteristics	Number of participants (%)
Age	
18–30	5 (27.8)
31–40	3 (16.7)
41–50	3 (16.7)
51–60	4 (22.2)
61 or above	3 (16.7)
Mean age	44.8
**Gender**	
Male	8 (44.4)
Female	10 (55.6)
**District**	
Hong Kong Island	7 (38.9)
Kowloon	6 (33.3)
New Territories	5 (27.8)
**Ethnicity**	
Chinese	18 (100)
**Highest education level attained**	
Postgraduate	3 (16.7)
Undergraduate	7 (38.9)
Higher diploma/Sub-degree	2 (11.1)
Secondary school	6 (33.3)
**Marital status**	
Single	5 (27.8)
Married	12 (66.7)
Divorced	1 (5.6)
**Employment status**	
Full-time	10 (55.6)
Part-time	3 (16.7)
Student	3 (16.7)
Homemaker	1 (5.6)
Retired	1 (5.6)
**Monthly income**	
HKD$20,000 or below	7 (38.9)
HKD$20,001–50,000	6 (33.3)
HKD$50,001–100,000	3 (16.7)
HKD$100,000 or above	1 (5.6)
Prefer not to say	1 (5.6)
**Religious affiliation**	
No religion	8 (44.4)
Buddhism	3 (16.7)
Taoism	2 (11.1)
Christian	3 (16.7)
Roman Catholic	2 (11.1)
**C-SWEMWBS score**	
25 or below	8 (44.4)
26–30	9 (50.0)
31–35	1 (5.6)
Mean score	25.9

C-SWEMWBS indicates Chinese Short Warwick-Edinburgh Mental Well-being Scale

**Table 3:** Summary of themes and their descriptions derived from think-aloud interviews

Theme	Description
Interview design and structure	Elements of design and structure that were valued, along with suggestions for refining presentation and instructions to improve validity.
Representation of items and levels	How participants understood and related to the different C-SWEMWBS items and levels.
Influences on decision-making	The personal and wider external factors that participants cited as influencing their decision-making.
Appropriateness of measures	Experiences of imagining states, comparing states, and difficulties in deriving utility values.

### Theme 1: interview design and structure

#### Appreciation for design

More than 80% of participants (15 out of 18) explicitly expressed satisfaction with the layout of tasks on the EQ-PVT platform, noting that the interface was user-friendly.

*“Um… Your design package is great. I can…er… use the mouse on the Zoom version to click*,* so even without a tablet or desktop*,* I can still participate. I think it’s already quite complete. Yes… Haha*,* so I’m very satisfied. I can cooperate with you in the test without feeling it’s too troublesome.”* (Male, 41).

#### Improving user experience

Five participants suggested minor refinements to the interface and visual presentation to enhance clarity. Four participants suggested representing levels with numerical values rather than verbal descriptors. Despite these suggestions, all participants were generally able to understand and complete the tasks as intended under the guidance of the interviewer.

*“Um*,* I think… if it’s good*,* then the use of scores to represent that state would be very intuitive. For example*,* it can be divided into… maybe the understanding of the word may not be the same*,* like sometimes*,* all of the times*,* often… so it’s a bit vague. But if we use scores*,* like 1*,* 2*,* 3*,* 4*,* 5*,* knowing their levels*,* it would be easier to… rank them in our minds… and then make choices based on those scores.”* (Female, 23).

#### Adapting to the process of time trade-off

During C-TTO tasks, five participants mistakenly clicked the non-preferred option. They required time to familiarise themselves with when questions began and ended, and the relationship between trade-offs and confirmatory statements.

*“I don’t quite understand (the confirmatory statement): Your answer suggests that in order to avoid spending ten years in that mental well-being state*,* you’re willing to give up 20 out of 20 years… but I don’t want to die so soon*,* so that doesn’t seem right. Maybe it’s not… Is it?”* (Male, 79).

#### Manageability of tasks

All participants generally considered both C-TTO and DCE to be manageable, though 12 participants saw C-TTO tasks as relatively more difficult due to its iterative nature. One participant suggested simplifying the process.

*“This kind of pressing buttons… actually*,* it’s not necessary.”* (Female, 58).

In contrast, the DCE tasks were perceived as requiring fewer steps, making them less cognitively demanding. All participants generally agreed that the number of items in a state was appropriate.

*“If there are too few*,* it’s not meaningful. So*,* seven seems to be the magic number.”* (Male, 58).

### Theme 2: representation of items and levels

#### Items as interconnected

The think-aloud process revealed that all participants perceived the C-SWEMWBS items to sufficiently represent MWB. Four participants expressed that the seven items have their unique meanings and are each important. Concurrently, despite unique differences between items, 11 participants noted that items are intrinsically linked, with the levels of one item influencing the experience of others.

*“Because if you are not optimistic about the future and you also have a bad assessment of your own abilities*,* it’s really difficult to find a way out and make choices. That’s how I feel.”* (Male, 41).

#### Interpreting the five-level structure

During the C-TTO tasks, all participants acknowledged that states in Life B were relatively inferior compared to the full MWB in Life A. However, seven noted that the highest levels of all C-SWEMWBS items did not necessarily represent the most desirable state. Of those seven participants, five suggested an optimal MWB state encompasses some items that are not “perfect”.

*“If you have negatives to compare*,* then you can appreciate the positives*,* right? I think it’s necessary to have… to have both positive and negative aspects to make a person more fulfilled. If everything is completely… perfect*,* meaning you can’t even see the negative aspects*,* it’s like not being very normal.”* (Female, 51).

#### Cognitive processing of items and levels

Participants adopted various cognitive strategies to understand the presented items and levels. For instance, 15 participants counted items at higher and lower levels, while 12 participants summarised items using binary statements like “good” and “bad” or “positive” and “negative”.

*“Okay*,* on the right side*,* the positive aspect is that I feel relaxed*,* can solve problems*,* and think clearly. But the negative aspect is that I’m not optimistic about the future and I won’t make friends.”* (Male, 33).

### Theme 3: influences on decision-making

#### The self and the wider society

All participants identified personal factors such as personality, age, and socio-economic background as influences on their preferences of MWB items and states, often leading to a higher weighting of certain items.

*“Because earlier*,* I mentioned that feeling relaxed is very important*,* so*,* well*,* it never happens*,* but these occurrences of feeling relaxed are very few*,* meaning there is at least some relaxation. So I think that’s better.”* (Female, 30).

Twelve participants highly valued “feeling close to other people” as enhancing tolerability in low MWB states. However, two participants expressed concerns about the potential burden on others if “feeling close to other people” were the sole item at a relatively high level within that state.

*“Ah*,* wow. I feel that even if I have people close to me*,* er… in this way*,* it’s a burden for them*.” (Female, 46).

While 10 participants did not explicitly acknowledge cultural influences on decision making in either task, two participants attributed their valuation of “ability to think clearly” to Hong Kong’s fast-paced lifestyle.

*“In other cultures*,* when unable to make decisions or having unclear thinking*,* those things may not be perceived as so important in their society.”* (Female, 23).

Among the ten participants with religious affiliations, those identifying as Roman Catholic or other type of Christian more likely incorporated religious beliefs into C-TTO decisions. Four participants articulated that their faith provided support during low MWB, leading them to adopt a more conservative stance during trade-offs.

*“We believe that God values us more than our own worth*,* so that’s the reason why I wouldn’t easily choose to give up on life.”* (Female, 35).

#### Eliminating harder-to-imagine states

Five participants expressed their preference towards relatable states, which are states they believe to be closer to what they have experienced in their daily lives or that they can envision themselves achieving. Two participants reported eliminating states they found harder to envision.

*“I would feel satisfied with a shorter period of time*,* it would be enough. The reason is*,* firstly*,* I have very rarely experienced such an ideal mental state*,* so it’s really difficult for me to imagine what that kind of life would be like.”* (Male, 41).

#### Avoidance of extreme levels

During the DCE tasks, eight participants exhibited a preference for states with more balanced levels, avoiding states with highest and lowest extremes.

*“If everything is a score of 3*,* it would be better than having some very bad and some very good situations. I tend to prefer everything being in the middle*,* where there’s nothing particularly good or particularly bad.”* (Female, 23).

### Theme 4: appropriateness of measures

#### Imagination of states

All participants generally managed to imagine the hypothetical states in both C-TTO and DCE tasks, despite expressing that MWB is seldom perfect. Seven participants, however, reported uncertainty about specific definitions of certain items, while still completing the tasks.

*“You didn’t specify how to define it*,* so I can consider my friends as being close*,* close to my family members*,* but being close to family members is different from being close to friends.”* (Female, 63).

Three participants found it challenging to imagine the varying levels of items described in MWB states.

*“Some scenarios have all three occurrences*,* sometimes*,* very few occurrences*,* sometimes… sometimes… often then sometimes*,* all of the times*,* that… So*,* um… it’s harder to think about.”* (Female, 30).

#### Failure to derive C-TTO values

Two participants (one with one instance and one with two) did not reach an indifference point for MWB states they viewed as worse than dead, resulting in values of negative infinity. Two instances involved the lowest state (1111111) and one a relatively low state (1212212). Even after trading all years of full MWB in Life A, they still preferred Life A (immediate death) to Life B (10 years of full MWB followed by 10 years in the target state), i.e., no finite amount made the options equivalent. Participants generally felt that the disutility of the low MWB states made immediate death preferable to living. The participant with two instances also noted that the contrast in Life B (full MWB followed by low MWB) would exacerbate suffering.

*“Because if I had a good first 10 years*,* but the last 10 years were bad*,* there would have been a big impact*,* and the pain in the last 10 years would have been exacerbated.”* (Male, 27).

## Discussion

This study is the first to explore the use of C-TTO and DCE for valuing C-SWEMWBS in Hong Kong. Four themes were identified to capture participants’ cognitive experiences during the think-aloud interviews: interview design and structure, representation of items and levels, influences on decision-making, and appropriateness of measures. While the results highlight areas for refining the design and valuation protocol, participants generally found both C-TTO and DCE tasks cognitively manageable, suggesting the feasibility of these methods for valuing C-SWEMWBS states in the Hong Kong general population.

### Theme 1: interview design and structure

Participants’ feedback provided insights into potential refinements. For instance, some suggested using numerical values to represent levels instead of words. While this could aid comprehension, it may compromise the purpose of the task by encouraging participants to calculate level sums rather than assigning their own subjective weights to C-SWEMWBS items and levels.

In addition, given the complexity of C-TTO exercises, interviewers could explicitly announce the beginning and end of each question, and transitions to the extended scenarios for states considered worse-than-dead. The C-TTO iterative process of reaching the point of difference was identified as a significant source of cognitive burden. One participant suggested directly determining the indifference point; however, this could introduce systematic inconsistencies and compromise data quality [29]. Previous studies found that participants who rushed through TTO tasks, reaching the indifference point within few iterations, more likely clustered their responses at a 0 value, inaccurately indicating that many states were equivalent to being dead [30, 31]. To ensure robust responses reflecting opportunity costs, it is essential to retain the step-by-step process for reaching the indifference point [13].

### Theme 2: representation of items and levels

Furthermore, patterns in participants’ representation of items and levels were observed. Non-monotonicity emerged for several participants completing the C-TTO and DCE tasks, where full MWB was not always deemed superior [32]. This phenomenon has been documented, though to a lesser degree, in our previous UK-based studies (i.e., one participant out of 14 in a think-aloud study and three out of 225 participants in the larger-scale valuation study) [2, 10]. Some participants interpreted full MWB as lacking challenging life experiences, which are elements that make life exciting [2]. Indeed, Confucian principles that are influential within traditional Chinese culture highlights the value of moderation, including the balance between different emotions, rather than the pursuit of optimal levels of individual functioning and happiness [33]. This may explain why, compared with previous UK-based studies, a significant proportion of participants in our study appeared to question whether full MWB necessarily represents the most desirable state [2, 10]. By extension, the cultural emphasis on moderation may have influenced the tendency for some Hong Kong participants to prefer states with more balanced levels, rather than states reflecting extreme highs and lows [33].

### Theme 3: influences on decision-making

Additionally, 12 participants prioritised the item “feeling close to other people”. The think-aloud process revealed that for many of these participants, this valuation was rooted in the importance of feeling close to family. This aligns with collectivist Chinese cultural values, which strongly emphasise filial piety, harmony, and respect for elders [34]. These values may also be, in part, reinforced by the geographical characteristics of Hong Kong, where the small land mass and highly connected transport system promote greater physical proximity to family. Several participants highly valued the item “ability to think clearly”. During the think-aloud process, two participants attributed this to the heightened pressure to succeed in the fast-paced culture in Hong Kong. Indeed, influenced by work values common to many other fast-paced Asian cities, workers in Hong Kong typically experience long working hours and strong pressures to fulfil rising job demands [35]. Concurrently, the think-aloud process revealed that a few participants displayed lexicographic preferences, prioritising certain attributes while overlooking others. Such behaviour, which violates the continuity axiom, has been documented in other health valuation studies [10, 36]. Without a think-aloud process, interviewers should remind participants to consider all items and levels carefully before deciding and monitor for overly quick response times, which may indicate decision heuristics or rushed responses [36].

### Theme 4: appropriateness of measures

Most participants could understand and imagine the MWB states derived from the C-SWEMWBS. While some expressed uncertainty about the exact definitions of certain items, this did not hinder task completion. Despite the C-SWEMWBS having been designed to be comprehensible to the Hong Kong public [7, 8], interviewers should provide participants with adequate opportunities to ask questions to ensure full comprehension of the C-SWEMWBS items before valuation tasks. Difficult-to-imagine states remain a challenge in health valuation studies, as they can introduce measurement errors [37, 38]. Echoing existing literature on EQ-5D-5 L health states, MWB states were considered harder to imagine if participants had rarely experienced them [39], leading to lower assigned values [38]. A few participants found the highest MWB state (5555555) unrelatable, thus required more effort to imagine. While an algorithm exists to flag highly uncommon SWEMWBS states [10], it is premature to exclude any deemed implausible in this study, as no clear consensus emerged on which MWB states were implausible [38].

Moreover, a minority of participants completing the C-TTO failed to derive utility values for states considered worse-than-dead. This echoed previous findings in the UK, where one of 14 participants failed to reach the indifference point for the lowest MWB state, yielding a utility value of negative infinity [10]. This was also observed in 1.38% of responses in a larger-scale preference elicitation study [2]. In this study, some participants expressed discomfort transitioning from a full MWB state to a low MWB state, suggesting that extending the lead time in Life B may not sufficiently increase its attractiveness to derive an indifference point [10].

### Limitations and future directions

Several limitations of this study should be noted. First, conclusions about cultural differences in decision-making between participants completing C-TTO and DCE tasks for MWB valuation remain limited. The think-aloud approach elicited some self-reported cultural factors in responses but did not provide a systematic approach for investigating how cultural factors influence decision-making [19]. Additionally, the study only included ethnically Chinese participants. While this reflects the majority (~ 92%) of Hong Kong’s population [40], recruiting individuals from ethnic minorities fluent in reading and speaking Cantonese proved difficult.

People with disabilities, who face an elevated risk of mental distress, represent a key group for optimising the inclusivity of valuation protocols [41]. While the C-SWEMWBS is a generic questionnaire for the general population, evidence suggests that individuals with mild intellectual disability may require additional attention from interviewers during DCE tasks due to their greater tendency to display lexicographic preferences [42]. Exploring the inclusivity of valuation protocols will ensure that findings reflect the MWB preferences of underrepresented groups. Nonetheless, this study demonstrates the feasibility of conducting a larger-scale preference elicitation study using C-TTO and DCE methods to derive a C-SWEMWBS value set for Hong Kong, enabling the estimation of MWALYs for cost-utility analysis of MWB interventions.

## Conclusions

This study explores the use of C-TTO and DCE methods for valuing the C-SWEMWBS in Hong Kong. Findings indicate that these methods are generally feasible and appropriate for eliciting MWB preferences from the general population. Establishing this suitability is important for accurate interpretation of responses and for informing the development of a Hong Kong‑specific C‑SWEMWBS value set. Such a value set will support robust cost‑utility analyses of mental health interventions and better informed, value‑for‑money decisions. 

## Supplementary Information

Supplementary Materials

## Data Availability

The datasets generated and/or analysed during the current study are not publicly available due to privacy concerns of sharing full interview transcripts. Study-related materials are available as supplementary materials or are available from the corresponding author on reasonable request.

## Abbreviations

C-SWEMWBS: Chinese Short Warwick‐Edinburgh Mental Well‐being Scale
C-TTO: Composite time trade-off
DCE: Discrete choice experiment
EQ-5D-5L: EuroQol five-dimensional five-level questionnaire
EQ-PVT: EuroQol Portable Valuation Technology
ICER: Incremental cost-effectiveness ratio
MWALY: Mental Well-being Adjusted Life Year
MWB: Mental well-being
QALYs: Quality-Adjusted Life Years
SD: Standard deviation
SWEMWBS: Short Warwick‐Edinburgh Mental Well‐being Scale
