# Supplementary material for: A think-aloud study exploring the application of composite time trade-off and discrete choice experiment methods for valuing the Chinese Short Warwick-Edinburgh Mental Well-being Scale (C-SWEMWBS)
Source: Health Qual Life Outcomes. 2025 Oct 23;23:108. doi: 10.1186/s12955-025-02433-4 (PMC12548184; doi:10.1186/s12955-025-02433-4)
Supplement: Supplementary file 1 — Supplementary Materials [file 12955_2025_2433_MOESM1_ESM.pdf]

Supplementary Information

Article title: A think-aloud study exploring the application of composite time trade-off and discrete choice experiment methods for valuing the Chinese Short Warwick-Edinburgh Mental Well-being Scale (C-SWEMWBS)

Journal name: Health and Quality of Life Outcomes

Authors:
Hei Hang Edmund Yiu, PhD[a*]; Ling Hin Chow, BPharm[a]; Cheri Cheuk Lam Au, MPhil[a]; Eunice Kehui Deng, MPharm[a]; Zimeng Zhao, B.A. & Sc.[a]; Yue Wei, PhD[a, b]; Kyung Jin Lee, MSc[a]; Caige Huang, BSc[a]; Yu Yang, MPhil[a]; Wei Kang, PhD[a, b]; Stavros Petrou, PhD[c]; Jason Madan, PhD[d]; Esther W. Chan, PhD[a, b, e, f*]

* Co-corresponding author

[a]Centre for Safe Medication Practice and Research, Department of Pharmacology and Pharmacy, LKS Faculty of Medicine, The University of Hong Kong, Hong Kong SAR, China

[b]Laboratory of Data Discovery for Health ($D^2$4H), Hong Kong Science and Technology Park, Hong Kong SAR, China

[c]Nuffield Department of Primary Care Health Sciences, University of Oxford, Oxford, United Kingdom

[d]Centre for Health Economics at Warwick, Warwick Medical School, University of Warwick, Coventry, United Kingdom

[e]Department of Pharmacy, The University of Hong Kong-Shenzhen Hospital, Shenzhen, China

[f]The University of Hong Kong Shenzhen Institute of Research and Innovation, Shenzhen, China

Corresponding authors:
Dr. Hei Hang Edmund Yiu
Centre for Safe Medication Practice and Research
Department of Pharmacology and Pharmacy
General Office, L02-56 2/F, Laboratory Block LKS Faculty of Medicine
The University of Hong Kong
21 Sassoon Road, Pokfulam
Hong Kong SAR, China
Tel: +852 3910 2269
E-mail: hheyiu@hku.hk

Prof. Esther W. Chan
Centre for Safe Medication Practice and Research
Department of Pharmacology and Pharmacy
General Office, L02-56 2/F, Laboratory Block LKS Faculty of Medicine
The University of Hong Kong
21 Sassoon Road, Pokfulam
Hong Kong SAR, China
Tel: +852 2831 5110
E-mail: ewchan@hku.hk

Table of Contents

Supplementary Information 1: C-TTO practice tasks 3

Supplementary Information 2: Debriefing questions for C-TTO 4

Supplementary Information 3: Debriefing questions for DCE 5

Supplementary Information 1: C-TTO practice tasks

Practice scenario	Description of the scenario
1	Imagine a mental well-being state characterised by a lack of confidence and self-esteem, one potential reason is your dissatisfaction with your living environment compared to that of your friends.
2 (a state better than the one described in the previous scenario)	Now, compared to your friends, your living environment is the best. As a result, you are in a mental well-being state characterised by happiness and high self-esteem.
3 (a situation worse than the one described in the first scenario)	Now, not only is your living environment the worst among the people you know, but you also have poor relationships with your friends. As a result, you are in a mental well-being state where your confidence and self-esteem are at their lowest point. You constantly doubt your role or ability in managing interpersonal relationships.

Supplementary Information 2: Debriefing questions for C-TTO

1. What do you think of the design of the task? Was it easy or difficult to complete?

2. Are there any aspects of the design that you think were done well or could be improved?

3. Was it easy to imagine the described states? Were there any states that were particularly easy or difficult to imagine?

4. Do you believe in the afterlife? Do you think your thoughts about death might have influenced your responses?

5. Do you think having a state of lower mental well-being would impact your life in any way?

6. If you were in the lowest mental well-being state or had mental disorders, do you think external support would help you cope? What steps would you take to cope with the situation?

7. If you were in a state of the lowest mental well-being, would you consider death as a possible solution?

8. Do you think Hong Kong culture, or the mix of Chinese and Western cultures, might have influenced your responses to this task?

9. Do you think your religion might have influenced your responses?

10. Do you have any other comments about the task that you would like to share?

Supplementary Information 3: Debriefing questions for DCE

1. What do you think of the design of the task? Was it easy or difficult to complete?

2. Are there any aspects of the design that you think were done well or could be improved?

3. Was it easy to imagine the described states? Were there any states that were particularly easy or difficult to imagine?

4. Was it easy to compare the states? Were there any pairs of states that were particularly easy or difficult to compare?

5. Did you think about the possible impacts of choosing one state to live in over another?

6. Do you think comparing seven items in a state is appropriate? Did it feel like too much or too little for you?

7. Do you think Hong Kong culture, or the mix of Chinese and Western cultures, might have influenced your responses?

8. Do you think your religion might have influenced your responses?

9. Do you have any other comments about the DCE tasks that you would like to share?

10. Overall, did you prefer the C-TTO or DCE task? Why?
